# A challenging entity: multiple sclerosis or collagen tissue disorders: A case series of 6 patients

**DOI:** 10.22088/cjim.8.4.321

**Published:** 2017

**Authors:** Raida Ben Salah, Yosra Cherif, Faten Frikha, Dammak Chifaa, Mouna Snoussi, Moez Jallouli, Sameh Marzouk, Mhiri Chokri, Zouhir Bahloul

**Affiliations:** 1 Department of Internal Medicine, Hedi Chaker University Hospital, Sfax-Tunisie.; 2 Department of Neurology, Habib Bourguiba University Hospital, Sfax-Tunisie.

**Keywords:** Multiple sclerosis, Collagen tissue disorders, Autoimmunity

## Abstract

**Background:**

Multiple sclerosis and other demyelinating processes are sometimes difficult to differentiate from the neurological involvement in autoimmune diseases. Distinguishing multiple sclerosis from other lesions due to autoimmune diseases is crucial to avoid unsuitable or delayed treatments.

**Methods:**

Charts of 6 patients diagnosed with mimicking multiple sclerosis between 1996 and 2014 were retrospectively assessed.

**Results:**

The mean age at diagnosis was 35±7 years. The most commonly neurological manifestation at onset was paraparesis due to transverse myelopathy and uni/bilateral optic neuropathy. All our patients suffered from recurrent episodes of optic neuritis with a mean lag time of 12 months. Other initial presenting neurological manifestations in our patients included ataxic gait and pyramidal syndrome. Systemic symptoms occurred a long time before or after their initial neurological presentation. All patients had numerous T2 hyperintense lesions in the periventricular white matter and spinal cord with contrast enhancement. The antibodies tests revealed the presence of significant amounts of anti-nuclear antibodies. The anti-phospholipid antibodies were negative in all patients. All patients were treated with corticosteroid therapy and neurological features were cleared in all cases.

**Conclusion:**

Multiple sclerosis, other myelitis and optic neuritis, are sometimes difficult to differentiate from CNS involvement in autoimmune disease. Indeed, the clinical presentation, immunological profile and MRI lesions may be similar.

**D**istinguishing multiple sclerosis (MS) from other lesions due to autoimmune diseases (AD) in patients presenting with inconclusive neurological symptoms is crucial to avoid unsuitable or delayed treatment. However, these may be the only early or even the first manifestations in systemic lupus erythematosus (SLE) and primary Sjogren’s syndrome (pSS), antiphospholipid syndrome and Behcet disease before the systemic features of the disease (1- 6). Numerous autoimmune and inflammatory diseases may masquerade as MS on MR imaging. Many patients with relapsing or progressive course and discharged with the diagnosis of MS were initially thought to have suffered from AD. The rate of AD patients whose pathology mimics MS at onset is unknown in the literature (2, 7, 8). Such a differentiation based on clinical status and neuroimaging may nevertheless be tricky. This diagnostic dilemma of MS versus AD is addressed in few studies (1-10). We report a case series of 6 patients who were diagnosed initially with probable MS with no evidence of AD or vasculitis for many years. Clinical, neuroimaging, immunological data and course disease are described in this report to warn for possible misdiagnosis of such lesions.

## Methods

We focused on 6 patients in the Department of Internal Medicine at Hédi Chaker University Hospital with atypical neurological manifestations and MRI abnormalities that did not initially fulfill the revised criteria for MS or collagen tissue disorders. These neurological lesions were associated with positive antinuclear antibodies (ANA) in all cases. Our patients were followed-up in Department of Neurology with the diagnosis of demyelinating disease until admission to the Department of Internal Medicine. 

All patients underwent complete physical examination and routine laboratory tests that included blood cell count, erythrocyte sedimentation rate (ESR), C-reactive protein, renal and liver tests, antinuclear antibodies (ANA), anti-phospholipid antibodies (aPL) Ig M and Ig G and urine analysis levels. Schirmer’s test and minor salivary gland biopsy were assessed in patients who had sicca syndrome. 

Cerebral and spinal MR imaging had been performed for each patient on admission and follow-ups. The presence of acute and/or chronic cortical/white matter lesions in the brain and spinal cord were also recorded. Their medical records were reviewed for follow-up and response to treatment. The definite diagnosis of AD or MS was based on international criteria. The diagnosis of pSS was based on the European Study Group on Classification Criteria for pSS, proposed by Vitali et al. (11). The diagnosis of connective tissue disease was based on the presence of antinuclear antibodies associated with systemic manifestations. The diagnosis of MS was established when the patient fulfilled the McDonald criteria for definite MS (12).

## Results

Clinical and imaging features of the patients are summarized in table 1. There were 6 patients in this study. The most commonly neurological manifestation at onset was paraparesis due to transverse myelopathy and uni/bilateral optic neuropathy. All our patients suffered from recurrent episodes of optic neuropathy with a mean lag time of 12 months.

**Table 1:** Characteristics of patients and course disease

**Patient/** **sex/age**	**Clinical manifestations**	**Systemic manifestations**	**Time lag between features and diagnosis**	**Immunological features**	**MRI abnormalities**	**Other investigations**	**Diagnosis**	**Treatment**	**Disease course**
F/28		Raynaud syndrome, hypothyroidism	3 years	ANA:1/320, positive AAT, Negative AcL, LA	Hyperintensities T2 and FLAIR periventricular lesions, corpus callosum	NORB	MS	Intravenous Methylprednisolone pulses Interferon	Stable
F/38	Paraparesis, Pyramidal syndrome	Arthralgias	15 days	ANA:1/320, Negative AcL, LA	Hyperintensities T2 of spinal cord, brainstem, cerebellum, corpus collasum		MS	Interferon	Stable
F/40	Paraparesis, pyramidal and Brown Sequard syndrome	Arthralgias	3 months	ANA:1/640, positive anti-centromeres, anti-PM-Scl, negative AcL and LA	Hyperintensities T1, T2 extended from 3^rd^ thoracic vertebra to 9^th^ thoracic vertebra.		Connective tissue disease	Corticosteroids	Complete recovery
F/19	Paraparesis, quadripyramidal and cerebellar syndrome	Visual blurring	1 year	ANA:1/1280, positive anti-SSA, anti-Scl70, negative AcL and LA	Hyperintensities T2 and FLAIR, periventricular lesions, corpus collasum and cerebellum, spinal cord lesions extended C3-C6, D1-D3, D7-D8-D10, L1-L2	Salivary biopsy: Chisholm 4, NORB, Ocular sicca syndrome	Sjogren syndrome	Corticosteroids	Complete recovery
F/42	Facial paresthesia, pyramidal syndrome	Visual blurring	6 years	ANA:1/640, positive anti-SSA	Hyperintensities T2 and FLAIR extended from C1 to C2	NORB, Chisholm 1	MS	Corticosteroids, Interferon	Remitting course
F/48	Facial paralysis, ataxia	Visual blurring and hallucinations, aphthous ulcers	1 year	ANA:1/640	Myelopathy	NORB Ocular sicca syndrome Chisholm 2	Sjogren syndrome	Corticosteroids, Cyclophosphamide	Partial recovery

Other initial presenting neurological manifestations in our patients included ataxic gait in case 6 and pyramidal syndrome in cases 3, 4 and 5. Systemic symptoms occurred a long time before or after their initial neurological presentation. All patients had numerous T2 hyperintense lesions in the periventricular white matter and spinal cord with strong contrast enhancement. No leptomeningeal involvement was observed in any patient. In this report, more detaited information about the 6 cases was given. 

**Figure 1:**
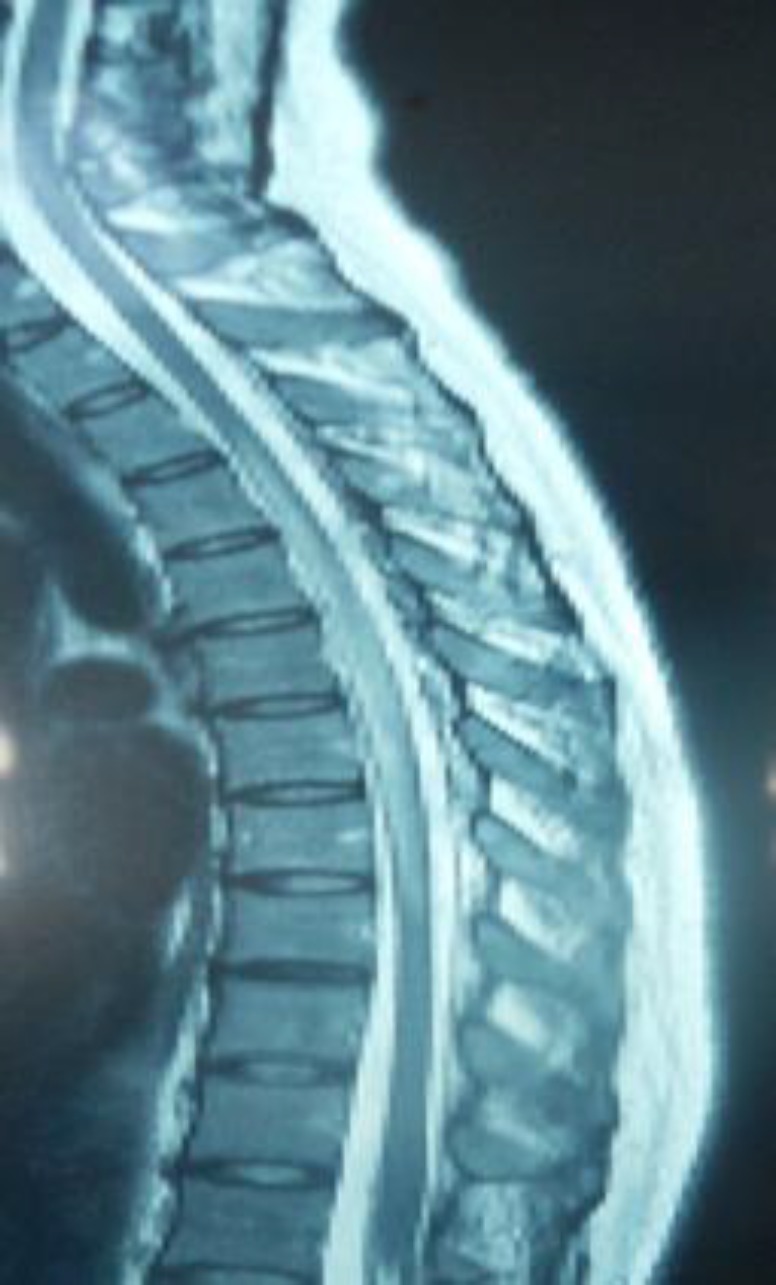
Cerebral and spinal MRI demonstrated isointensities on T1 weighted images, T2 hyperintensities extending from the 3^rd^ to the 9^th^ thoracic vertebrae with a strong enhancing Gadolinium signal

**Case 1: **A 28-year-old woman was admitted in August 2010 to the Department of Neurology complaining of headache, paresthesia and progressive paraparesis for 1 month. Neurological examination revealed no sensory or motor disturbances. Cerebral and spinal MRI was performed and showed T2 and FLAIR hyperintensities involving the pons, periventricular white matter and corpus callosum without contrast enhancement (figure 1). 

Visual-evoked potentials were normal. Thus, she was diagnosed with MS and was given 5 intravenous methylprednisolone pulses and interferon β and remained asymptomatic during 1 year. In the second year of interferon therapy, she described Raynaud syndrome with no other systemic manifestations. She was admitted to the Department of Internal Medicine. The MRI revealed T2 hyperintensities of left cerebellar peduncle, corpus callosum and periventricular white matter. The spinal MRI was normal. The immunological profile revealed simultaneously positive ANA:1/320, positive antithyroglobulin antibodies and negative anticardiolipin antibodies. The thyroid test showed hypothyroidism. Like her first episode, her paraparesis gradually improved with intravenous corticosteroid pulses. The patient was discharged 2 days with the diagnosis of MS without any neurological disorder. A follow-up MRI 6 months later showed residual lesions in the same areas. 

**Figure 2:**
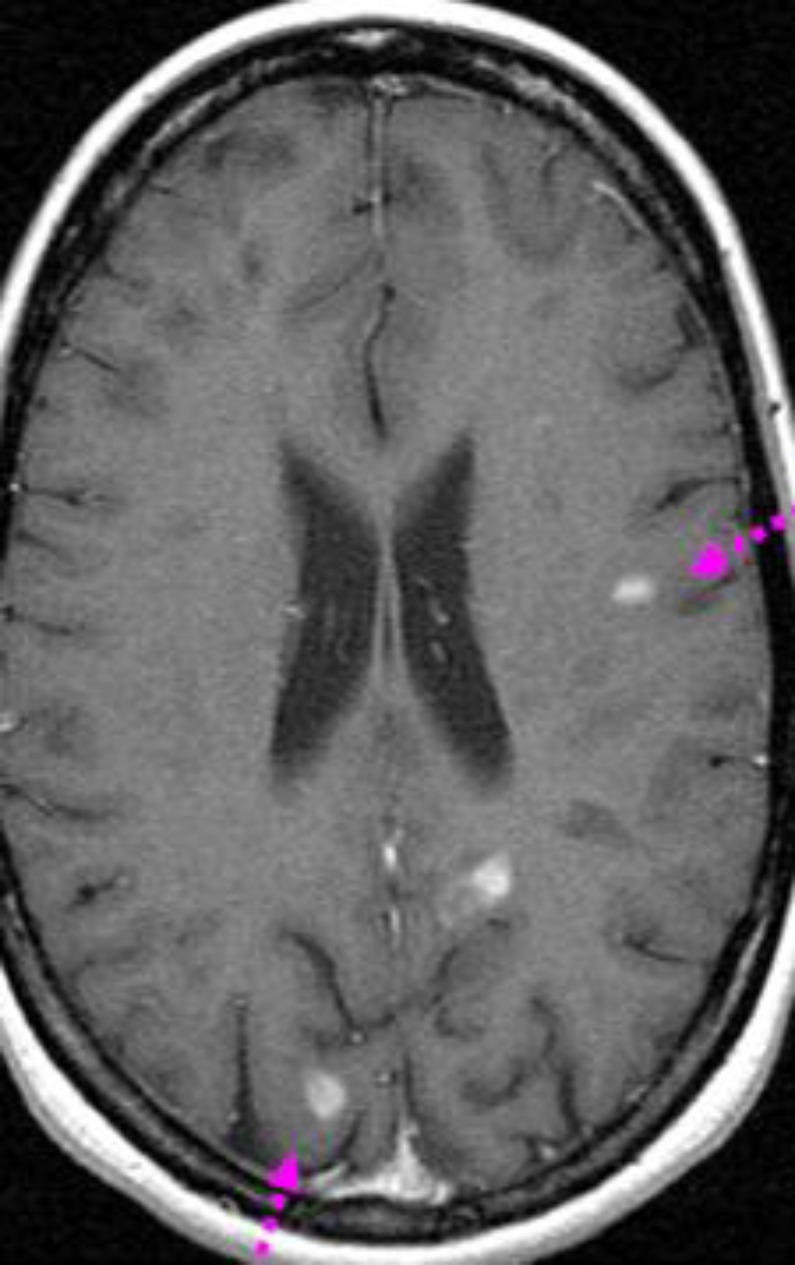
Cerebral MRI enclosed multiple T2 hyperintensities of brainstem, cerebellum, corpus collosum and periventricular white matter

**Case 2: **A 38-year-old woman with a long history of bronchiectasis was admitted with paraparesis and arthralgia of knee, ankle and metacarpophalangeal joint for 2 weeks. The physical examination showed paraparesis, right pyramidal syndrome, bilateral Babinski reflex and motor deficit of the right upper and lower limbs. She also complained of left blurring vision. Cerebral and spinal MRI enclosed multiple T2 hyperintensities of spinal cord, brainstem, cerebellum, corpus collosum and periventricular white matter (figure 2). Routine laboratory tests were within the normal range. All serological profiles of neurotropic viruses were negative. Moreover, ANA: 1/230 were positive and antiphospholipid antibodies and lupus anticoagulant were negative. The diagnosis of MS was established based on McDonald criteria for definite MS. She was treated by interferon therapy. The disease course was uneventful. She discontinued her treatment and regular outpatient check-ups 2 years later.

**Figure 3 (A, B).**
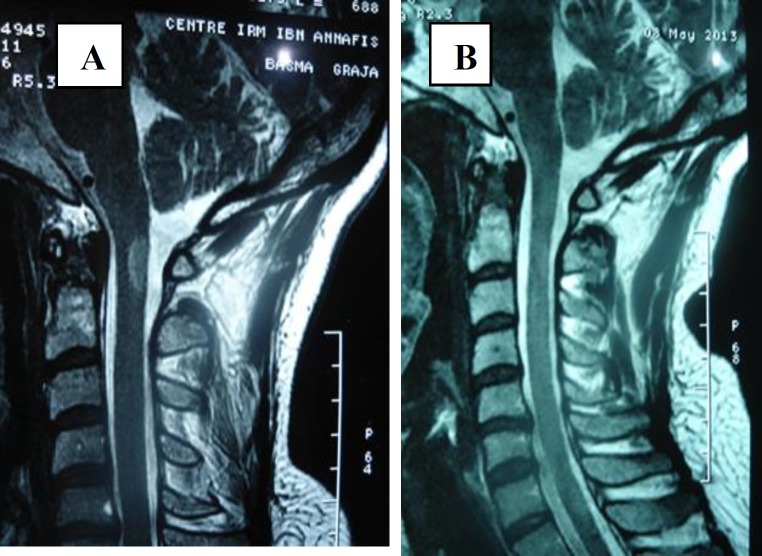
Cerebral and spinal MRI demonstrated isointensities on T1 weighted images, T2 hyperintensities extending from the 3^rd^ to the 9^th^ thoracic vertebrae with a strong enhancing Gadolinium signal

**Case 3: **A 40-year-old woman had a history of inflammatory arthralgias and arthritis for many years occasionally treated with non-steroid anti-inflammatory drugs. Three months before her admission to Department of Neurology she developed progressive paraparesis and bilateral motor deficit. Physical examination revealed quadripyramidal syndrome, Brown-Sequard syndrome, ataxic gait and spastic paraparesis without other neurological disorders and systemic manifestations. Cerebral and spinal MRI demonstrated isointensities on T1 weighted images, T2 hyperintensities extending from the 3^rd^ to the 9^th^ thoracic vertebrae with a strong enhancing gadolinium signal (figure 3A). 

The examination of cerebrospinal fluid showed increased albumin level. Screening for infectious disease and neoplasm was negative. Immunological tests disclosed positive ANA: 1/640, positive anti-centromeres and anti-PM Scl both in serum and cerebrospinal fluid. Antiphospholipid antibodies and lupus anticoagulant were within the normal range. A repeated physical examination did not detect symptoms of systemic scleroderma or collagenous tissue disorder. The patient was treated with 3 intravenous methylprednisolone pulses linked with a high regimen of corticosteroid: 1 mg/kg/day during 6 weeks then tapered till 10 mg/day. The disease course was uneventful, and neurological disturbance improved gradually. Approximately, one year after the diagnosis, MRI was again evaluated and it was normal (figure 3B).

**Figure 4 (A, B).**
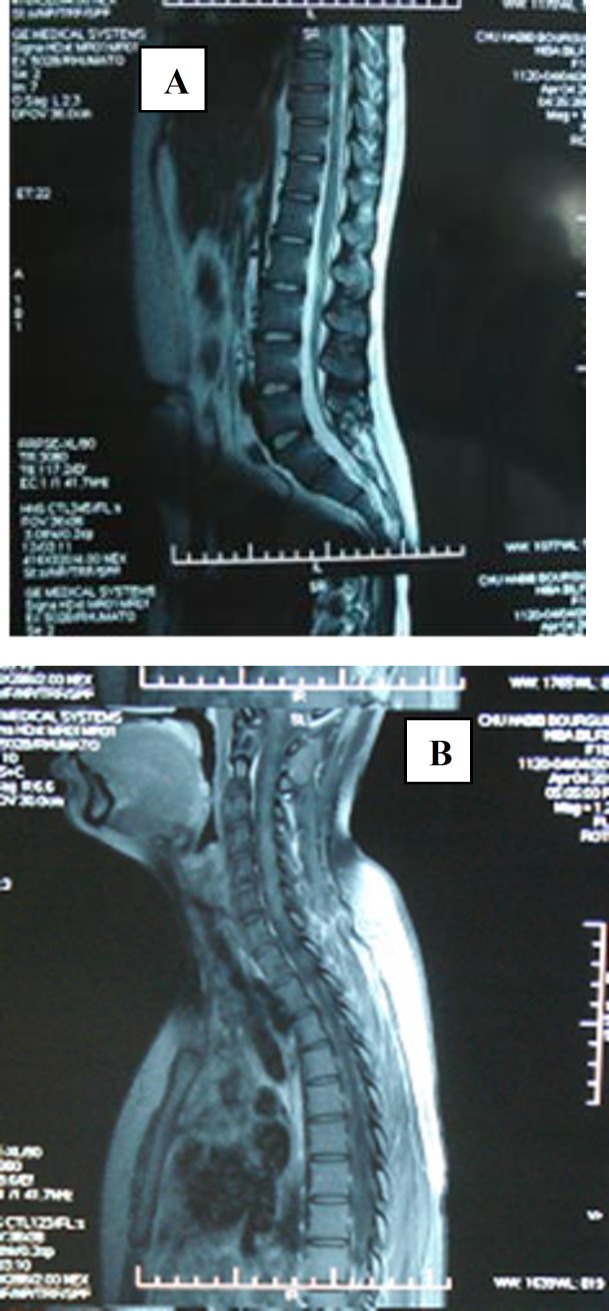
The spinal cord MRI revealed else increased T2 signal extended from the 3^rd^ cervical vertebrae to the 10^th^ thoracic vertebra and from the 1^st^ lumbar to the 2^d^ lumbar vertebrae

**Case 4: **A 19-year-old woman with no preexisting medical history presented in April 2012 with progressive spastic paraparesis, ataxia and bilateral pyramidal syndrome. Full blood tests were normal. Cranial MRI showed increased intensities on T2 and FLAIR weighted images in the periventricular white matter, corpus callosum, brainstem and cerebellum. The spinal cord MRI revealed another increased T2 signal extended from the 3^rd^ cervical vertebrae to the 10^th^ thoracic vertebra and from the 1^st^ lumbar to the 2d lumbar vertebrae (figure 4A). 

Visual-evoked potential was a characteristic of bilateral retrobulbar optic neuritis. She was diagnosed as having MS and was treated with intravenous methylprednisolone pulses. She remained well for the next 12 months, with partial recovery of neurological disorder. As the immunological screening showed positive anti-nuclear antibodies: 1/640, positive anti-SSA, she was referred to the Department of Internal Medicine to exclude a systemic autoimmune disease. 

The physical examination was normal except bilateral pyramidal syndrome and ataxia. The patient particularly denied symptoms of dry eyes or mouth. Full blood tests remained normal. 

The ophtalmoloical examination was normal and showed no sicca syndrome. The revised ANA was positive: 1/1280. Anti-Scl70 was positive. Minor salivary biopsy revealed Chisholm grade IV. Our patient did not fulfill the criteria of Sjogren’s syndrome. Thus, we decided to monitor our patient with no other treatment. In August 2012, she presented a hypokalemia (potassium level at 3.2 mmol/l) with no clinic features. Cerebral MRI in December 2012 was performed to look for changes suggestive of demyelination and showed the same increased T2 signals in periventricular white matter, brainstem and corpus callosum with no new lesions in other areas (figure 4B). 

Cerebrospinal fluid analysis was repeated and demonstrated positive immunoglobulin G oligoclonal bands. Ophthalmological examination showed bilateral punctate keratitis. Over this year, she developed a recurrent episode of ataxia and spastic paraparesis. Relapsing neurological manifestations, optic neuritis and neuroimaging seem to favor the diagnosis of MS. 

However, our patient fulfilled the diagnostic criteria of Sjogren’s syndrome. Since December 2013, neurological relapse did not recur. Then, she was not treated and she continues to be asymptomatic. 

**Figure 5:**
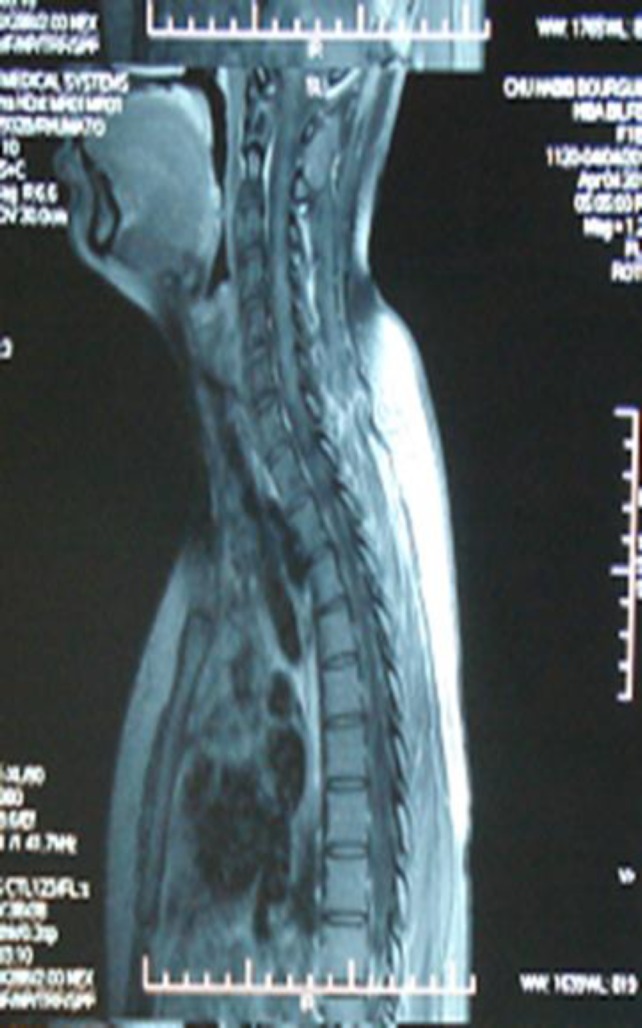
The spinal cord MRI revealed T2-weighted images a homogeneous hyperintensity was found extending along the 1^st^ and 2^nd^ cervical vertebrae and demonstrated moderate gadolinium enhancement

**Case 5: **A 42-year-old female was monitored by a neurologist and suffered from left sided hemiparesthesia, muscle weakness of the left upper limb and bilateral visual blurring in 2007. Visual evokated potentials enclosed bilateral optic neuritis. On T2-weighted images, a homogeneous hyperintensitiy was found extending along the 1^st^ and 2^nd^ cervical vertebrae and demonstrated moderate gadolinium enhancement (figure 5). All the laboratory data including routine tests, serological and immunological tests were within normal range. Our patient was diagnosed with MS. She was treated by corticosteroid. The neurological disorders were improved. Neuro-imaging showed no lesions 6 months after treatment. She presented with several relapsing remitting optic neuritis and forevermore treated with corticosteroids with partial improvement. She was admitted to our department in July 2013 for right facial paresthesia with positive ANA: 1/640, positive anti-SSA. A thorough neurological examination was normal. She did not exhibit extraglandular or systemic features of collagenous tissue disease. The ophthalmological examination showed sicca syndrome and minor salivary biopsy disclosed Chisholm grade 1 sialadenitis. Our patient did not fulfill the diagnosis criteria of Sjögren’s syndrome. She was not treated and still followed-up in the outpatient department with relapsing bilateral optic neuritis. 

**Case 6: **A 48-year-old woman was admitted to the Department of Internal Medicine with ataxia, blurred vision, visual hallucinations and in coordination of the right limbs with no dysarthria. Ten months previously she had developed severe headaches and paraesthesia of the right face and limbs and right facial nerve paralysis, with incomplete recovery under physiotherapy. She reported recurrent oral aphthous ulcers but no dry eye/mouth syndrome or other systemic features. Physical examination revealed an ataxic gait with an extensor right plantar response. 

**Figure 6:**
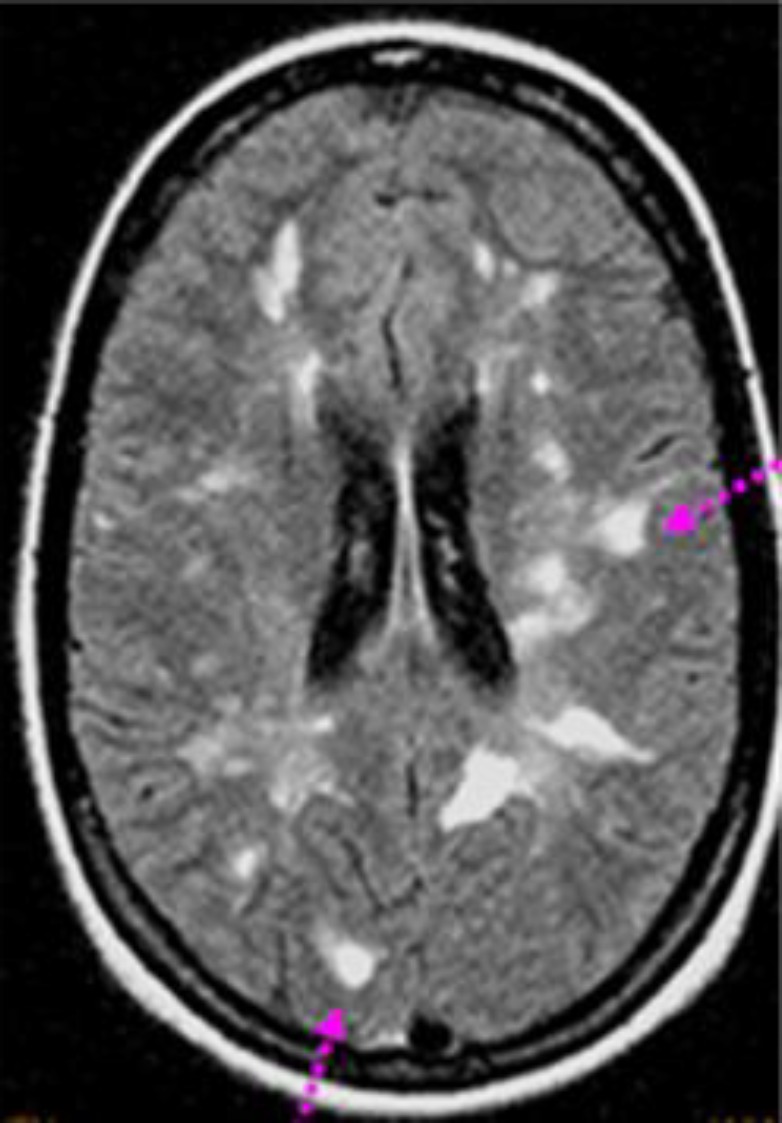
Cerebral MRI showed multiple enhancing lesions of bulb, cerebellar peduncles and extensive to the cervical spinal cord

Visual evoked responses showed prolonged latencies from each eye compatible with bilateral retrobulbar optic neuritis. Cerebral MRI showed multiple enhancing lesions of bulb, cerebellar peduncles and extensive to the cervical spinal cord (figure 6). The lumbar cerebrospinal fluid was normal. Different serological tests including HIV, hepatitis virus B and C, HSV, CMV and EBV were negative. The immunological profile showed positive AAN:1/640. Anticardiolipin antibodies, lupus anticoagulant and cryoglobulinemia were within the normal range. Ophthalmological examination disclosed a sicca syndrome. Accessory salivary gland biopsy showed grade 2 sialadenitis. Our patient was diagnosed with Sjogren’s syndrome.

She was treated with high dose intravenous methylprednisolone and monthly cyclophosphamide pulses with marked neurological improvement and no recurrence within 2 years later. Still she had chronic residual lesions with T2 hyperintensities on periventricular areas.

## Discussion

We report 6 female patients presenting with myelitis, few systemic features and positive immunological profile. Today the diagnosis of MS or autoimmune disease with neurological involvement is mainly based on clinical symptoms, paraclinical findings and cerebral MRI. Due to similar clinical and neuroradiological features, cerebral and spinal lesions are prone to be misdiagnosed (2, 6, 7, 8). The spectrum of diseases potentially leading to a wrong diagnosis includes infections, vasculitis and ADs (8). Spinal cord involvement is more commonly described in MS patients, but it has also been reported in patients with different ADs (1, 2, 8, 9). Transverse myelopathy in systemic lupus erythemousus (SLE) is reported in 1-2 % of SLE patients (1, 13) with gradually progressive or subacute or acute onset. There are also case reports of primary Sjögren syndrome, Behçet’s disease and antiphospholipid syndrome with acute transverse myelopathy as the initial manifestation (1, 3, 6, 7, 14, 15). The time lag between diagnosis of SLE, Sjögren’s syndrome or other AD and the onset of transverse myelopathy varied widely (4, 15). Our series showed long segment myelopathy in cases 3 and 4 that was suggestive of Devic neuromyelitis but the anti-NMO and aquaporin-4 antibodies were negative and their MRI findings never fulfilled the criteria for MS. 

The relapsing clinical course of MS may mimic that of ADs (4, 8). Five of our patients had a remitting course (cases 1, 2, 4-6). Case 3 presented with only one episode of myelitis with positive anti-centromere antibodies that responded well to corticosteroid therapy with no relapse. In all cases, myelitis was the first manifestation which may be associated or not with optic neuritis. 

In rare cases, a demyelinating disorder can also initiate ADs. Besides, the clinical history in patients with MS is not usually conclusive (1). Atypical associated symptoms and positive immunological profile led to the wrong diagnosis of collagen tissue disorders in our cases 1, 2 and 4. In our series, case 1 had an interval of two years between transverse myelitis and Raynaud syndrome. In addition, cases 2 and 3 presented with previous arthralgias for many years before the diagnosis as far as case 6 that simultaneously developed prominent sicca syndrome. It is noteworthy that our patient (case 4) did not relate xerostomia or xerophthalmia at the onset of the initial neurological symptom. Primary Sjögren’s syndrome was suspected only when positive ANA with anti-SSA and anti-SSB antibodies were found. 

An undoubted distinction between MS, autoimmune disease, collagen tissue disorder can be tricky, because clinical, laboratory features, cerebrospinal fluid findings and even MRI lesions may be indistinguishable and not helpful. In SLE and Sjögren’s syndrome with neurological involvement, oligoclonal band analysis is positive in up to 50 % of patients (16, 17). In our series, 4 patients displayed oligoclonal band positivity. Otherwise, optic neuritis can predict the diagnosis of MS. Although, it may be associated with several ADs (4, 18, 19). It occurs in only 1 % of patients with SLE (1, 4, 18, 19) and primary Sjögren’s syndrome (1). Four among our 6 patients had recurrent unilateral or bilateral severe optic neuritis. None of the patients had a wellestablished setting of AD during optic neuropathy. Only 1 of our patients (case 6) had simultaneous sicca symptoms in the ophthalmological examination.

While conventional MRI is frequently employed for acute neurological deficits especially in MS (18, 19), central nervous system lesions cannot be distinguished from inflammatory conditions in all of the cases (6, 20). Nevertheless, these conventional techniques alone are not sufficient to recognize all of these conditions, especially when lesions are untypically located or unexpectedly changed over time. Myelopathy for instance may be falsely interpreted as an infarct, or misinterpreted by MRI as MS (6, 7, 20). An atypical distribution of lesions in ADs is scarcely described. The most common abnormalities in addition to myelopathy in cerebral MRI were multiple T2-hyperintense lesions, which prevailed in the periventricular cerebral white matter. The spinal cord involvement pattern was imperceptible in different patients with MS or other ADs: with increased T2 signal intensity and homogeneous gadolinium enhancement in acute episode and partial or complete resolution of the lesions on follow-up MRI (6, 8, 21, 20) like all our cases. 

The prevalence of antinuclear and antiphospholipid antibodies has been reported to widely vary the patients with MS (2-44 %) (5, 9, 22-24). Karussis D et al. reported 20 among 100 cases of MS with positive anticardiolipin antibodies (25). Moreover, anti-SSA antibodies can be detected in MS with a rate range between 2-15% (22). No patient in our series presented with positive antiphospholipid antibodies but antinuclear antibodies were positive in all cases even in these with atypical MS and systemic features. Therefore, one has to keep in mind that the persistence of high ANA titers furthers collagen tissue disorder. The prognosis depends on the prompt diagnosis, extent of spinal cord involvement, and prompt treatment with corticosteroid pulses, and other immunosuppressive drugs (3, 5, 14, 26). Treatment with intravenous methylprednisolone associated with cyclophosphamide seemed to be most effective in typical transverse myelopathy and optic neuritis related to SLE, Sjögren’s syndrome and other ADs (3, 6, 19, 13, 26).

Our patients (cases 3, 4 and 6), who had clinical profile mimicking MS, showed a favorable response to corticosteroids. Only one patient (case 6) received monthly cyclophosphamide pulses. During a close follow-up, patients may develop systemic symptoms unexpected in MS, like arthralgias, arthritis and sicca syndrome leading to the final definite diagnosis. 

In summary, we wanted to show, first, that despite tremendous advances in neuroimaging, distinguishing the presentation of MS and other inflammatory pathologies with neurological involvement and typical extensive transverse myelitis remains an important diagnostic challenge, and second, that positive immunological profile out of systemic manifestations should not be taken in consideration in diagnosing connective tissue disorders or vasculitis. Nonetheless, it might be strongly considered in the course of disease because patients may meet later the diagnosis criteria of ADs. As a result, careful clinical follow-up of such patients is recommended. 

## Conflict of Interests:

None declared.
